# A 20-Country Comparative Assessment of the Effectiveness of Nutri-Score vs. NutrInform Battery Front-of-Pack Nutritional Labels on Consumer Subjective Understanding and Liking

**DOI:** 10.3390/nu15132852

**Published:** 2023-06-23

**Authors:** Jun He, Marco Francesco Mazzù, Angelo Baccelloni

**Affiliations:** 1 Department of Business and Management, Luiss University, 00197 Rome, Italy; jhe@luiss.it; 2 Department of Business and Management, Luiss Business School, 00162 Rome, Italy; 3 Department of Business Administration, Frank J. Guarini School of Business, John Cabot University, 00165 Rome, Italy; angelo.baccelloni@johncabot.edu

**Keywords:** NutrInform Battery, Nutri-Score, subjective understanding, objective understanding, liking, front-of-pack nutritional labels (FOPLs), food policy, consumer behavior, European Union

## Abstract

The incidence of overweight and obesity has generated significant concerns among European consumers and institutions. As part of a set of measures undertaken, the European Union (EU) called for one harmonized mandatory front-of-pack nutritional label (FOPL) to improve consumer food nutritional knowledge and encourage healthier and more informed food choices. Different types of FOPLs, ranging from nutrient-specific labels—such as the NutrInform Battery—to summary labels—such as the Nutri-Score—have been developed and introduced in different markets, reporting different degrees of effectiveness in terms of understanding. The aim of this study is to provide actionable insights by analyzing a specific part of the complex consumers’ decision-making process in food when aided by FOPLs. Adopting a between-subject experiment on a sample of 4560 respondents in 20 EU member countries, the study compares the consumer subjective understanding and liking of two labels currently under examination by the EU bodies, the NutrInform Battery and the Nutri-Score. At an aggregated level, the results show that NutrInform Battery is more effective than Nutri-Score in improving consumer subjective understanding and leads to a higher liking towards the label. A detailed by-country analysis highlights either a superiority or a parity of NutrInform Battery for subjective understanding and liking. Theoretically, this study, through a large panel of respondents, adds the fundamental perspective on subjective understanding, complementing the findings of extant research on objective understanding, and further clarifies the role of liking as a complementary element in the food decision-making process toward heathier and more informed food choices. This might be of significant relevance in providing additional evidence that can be used by policymakers in their attempt toward the selection of a uniform FOPL at EU level.

## 1. Introduction

According to a report by the World health Organization (WHO) [1], almost 60% of adults in Europe and around 30% of children are affected by overweight and obesity, a factor highly associated with non-communicable diseases. In 2020, the EU “Farm-to-Fork” strategy was launched as one of the initiatives to improve the nutritional status, public health, and environmentally friendly food consumption in Europe [2], also promoting the utilization of a harmonized mandatory FOPL across European countries as one of the solutions to increase consumer understanding of food nutritional knowledge and stimulate healthier food reformulation among food marketers [3].

Front-of-pack nutritional labels are characterized by understandable and salient nutrition information presented in the front pack of pre-processed foods [4]. The placement of nutrition labels in the front pack is reported to increase consumer awareness of nutrition information [5], and their presence is cognitively processed by consumers as an indicator for food evaluation [6], according to which the perceived nutrition quality of healthier food products increases, while the perceived healthiness of less healthy food decreases [7,8,9]. In turn, FOPLs enable consumers to make healthier choices [6,9,10].

Several different FOPLs have been adopted in the past and classified according to an EU taxonomy on the basis of their informative and directive strengths, as “nutrient-specific labels” and “summary labels” [11]. Nutrient-specific labels, such as the “NutrInform Battery”, provide consumers with specific and nondirective numeric information. Summary labels, instead, might include endorsement logos, such as the “keyhole” label, widely used in Nordic countries, “warning labels”, as promoted in Latin America [12,13], or graded indicators, such as the “Nutri-Score”, already adopted in some European countries.

In the current situation, where a mandatory harmonized label is pursued across the EU [3], it is critical to understand which type of FOPL, compliant with Regulation (EU) No. 1169/2011, will be more suitable for Europe-wide use [14,15]. A guiding element to support the decision is the analysis of the implications of FOPL utilization on food decision-making, where the role of consumer understanding for an appropriate use of nutrition labels is central [16]. Grunert and Wills’ [17] theoretical model, one of the most widely used in recent FOPL literature, categorizes consumer understanding into objective and subjective. The first one is “whether the meaning the consumer has attached to the label information is compatible with the meaning that the sender of the label information intended to communicate”; the second one focuses on “the meaning the consumer attaches to the perceived label information and also covers the extent to which consumers believe they have “understood” what is being communicated”.

Recent studies have shown that consumers refer to FOPLs and make significantly different decisions when they need to put food together as a combined diet compared to when FOPLs are absent [8,18,19,20,21]. These findings reinforce the fact that, in agreement with the Grunert and Wills model [17], the comprehension of the consequences of FOPLs’ usage by consumers cannot be oversimplified as a sum of individual choices based only on objective understanding. When assessing consumers’ internal response to FOPLs before their behavioral intentions, subjective understanding has a fundamental complementary role to objective understanding. In addition, FOPL liking [22,23], a variable that runs parallel to understanding, should be considered wherein “label that is liked can lead to a more positive evaluation of the product even when it is not understood” [17].

In this research, we propose that it is essential to study from different angles how consumers understand FOPLs in different ways to understand a complex and multi-faceted process as the one related to food choices. We selected two front-of-pack labels—NutrInform Battery and Nutri-Score—both of which were developed by EU member countries while posited in the different FOPL categories of nutrient-specific labels and summary labels. Thus, we compared how the NutrInform Battery and the Nutri-Score affect consumer subjective understanding and liking.

Specifically, the NutrInform Battery [15] was developed in Italy between 2018 and 2019 in collaboration with four government ministries (Economic Development, Agriculture, Health, and Foreign Affairs) and with technical and scientific support from two government Research Institutes (ISS National Institute of Public Health and CREA Food and Nutrition Research Center). The NutrInform Battery, categorized as nutrient-specific informative FOPL, aims to assist and empower consumers to make conscious dietary decisions based on general daily nutritional requirements. This labeling system provides consumers with information regarding the number of calories, sugar, fats, saturated fats, and salt per serving, which is based on the recommendations outlined in the official Italian dietary guidelines known as “Reference Assumption Levels of Nutrients and Energy for the Italian population” (LARN). Furthermore, the NutrInform Battery incorporates an easily interpretable battery symbol. This visual symbol has proven not to be confounding for consumers [24] and enables them to visualize the energy level and main nutrients present in a serving of the product concerning their daily reference intakes (RIs).

Nutri-Score [25], which was primarily based on British Food Standards Agency Nutrient Profiling System and subsequently modified by the High Council for Public Health to align with French dietary guidelines, was implemented in France in 2017. The Nutri-Score, categorized as a summary label, aims at evaluating multiple elements through an algorithm. The algorithm assigns grades to food products based on several criteria using 100g as a standardized unit of measurement. These criteria include energy, saturated fat, sugar, sodium, fiber, protein, and proportion of fruit/vegetables/nuts. Based on the final grades, Nutri-Score presents consumers with five levels, ranging from dark green to red (represented by A to E), with dark green representing the healthiest choice and red E as the least heathy choice. (The examples for The NutrInform Battery and Nutri-Score are provided in Figure S1).

Nutri-Score has been extensively studied over the past years [25,26,27,28,29] by utilizing the lens of objective understanding; extant research confirmed its consistent superior effectiveness in different countries [9,30,31,32,33,34], mainly measuring the relative ranking the nutrition quality of a set of products under the same category/within the same categorical set. While in this vein, it is assumed that consumer usage of FOPLs is restrained in the context of individual choices among a set of food in the same categories, researchers emphasized the potential of objective understanding in improving consumer food preferences [27,28]. Other studies showed that, more in general, FOPLs have limited or no significantly different effectiveness in changing consumer food options at the point of sale, where foods of different nutritional values in the same category are arranged in similar places for comparison [8,18,19,20]. Despite the limited effect of FOPLs in the point of purchase, recent studies found significant results in terms of consumer use of FOPLs as instrumental tools in more realistic food consumption simulation, based on a few food choices bounded as an entire lunch [21]. Although Pettigrew et al. [9] recently reported that Nutri-Score increases consumer preference for more healthy food, as well as increasing aversion for less healthy food, their earlier studies reported that different FOPLs have limited significant differences in affecting final food choices [34].

Those results, while consistent among each other, disregard the perspective on subjective understanding and liking, fundamental aspects in the food decision-making model [17]. Previous researchers emphasized that consumer knowledge should be considered beyond objective understanding. Specifically, Grunert and Wills [17] explained that exposure to the label reinforces the search for front-of-pack nutritional label information. However, to move consumers to behavior, the information should be perceived (in a conscious or unconscious way), subsequently leading to understanding. The authors also highlight the role of liking of the label, not necessarily linked to the understanding, but which might have an impact on the actual utilization of the FOPL, even in cases when the label is not understood. This complex decision-making process cannot be over-simplified when assessing how consumers might use or not the front-of-pack nutritional labels to make their informed choices toward healthier diets.

In this light, previous studies have shown the superior performance of the NutrInform Battery in improving consumer subjective understanding and liking in several EU countries, including France, Germany, Greece, Italy, Portugal, Romania, Spain, Slovenia, the Netherlands, and Poland [11,15,24,35].

In response to the European Commission’s call for a harmonized front-of-pack label to apply across the European countries, we reorganized past studies on subjective understanding across 10 EU member countries as secondary dataset and enlarged the sample by further incorporating an additional 10 EU countries to compare the effectiveness of NutrInform Battery and Nutri-Score in terms of consumer subjective understanding and liking. We then make the following two hypotheses:

**Hypothesis** **1:** *NutrInform Battery is more effective than Nutri-Score in improving consumer subjective understanding of FOPLs in the European Union.*

**Hypothesis** **2:** *Consumers have a higher liking level for NutrInform Battery than Nutri-Score in the European Union.*

## 2. Materials and Methods

Studies were designed as a between-subject online experiment, with one nutrient-specific label (NutrInform Battery) and one summary label (Nutri-Score) used in the experiments as independent variables and attached to the same food categories. For each category, mock packages were used to avoid confounding effects or bias derived from association with brands [24,36]. Subjective understanding and liking were identified as the two dependent variables for the study. Subjective understanding was measured by pre-validated scales on the three constructs of comprehensibility [37], help to shop [37], and complexity reduction [37], while liking was based on Allen and Janiszewski [38]. The specific measures are shown in Table 1.

All customers of the cumulated sample were exposed to the same methodology in terms of structure of the questionnaire, data collection, and stimuli [11,15,24,35]. The primary data collection was carried out across the remaining 17 EU countries, together with the previous 10 countries from secondary data, to achieve a complete understanding of all EU member countries. The questionnaires were administered, with the same structure, via the Prolific online survey platform, a recently established international web panel provider that combines high recruitment standards and proper response rate, reliability, and high replicability of studies [39]. Data collection was not displaying a sufficient number of valid responses from Bulgaria, Croatia, the Republic of Cyprus, Lithuania, Malta, Slovakia, and Luxemburg. As a result, we excluded those countries from the analysis and reported the results of the remaining 10 EU member countries with a sufficient number of respondents.

The collective perspective provided by the newly collected data from these 10 countries and the previous 10 countries provides a robust representation in terms of internal validity to observe specific steps of food decision making [4]. The overall sample includes a representation of countries characterized by different levels of obesity and overweight. For example, in 2019, a Eurostat (https://ec.europa.eu/eurostat/statistics-explained/index.php?title=File:Share_of_overweight_population_by_sex_and_age,_2019_(%25).png accessed on 25 May 2023) study showed that while overweight display an average of 52.7% in the EU population over 18 years, individual countries in the study panel perform differently, ranging from Italy at 45.7% to the Czech Republic at 60.0%, with a mean value across 20 countries of 54.5% (SD = 0.04). (The detailed obesity statistics for all countries are available in Supplementary Materials—Table S2).

All participants were required to fill in the consent form before the survey, were informed of the role of FOPLs, and then completed the survey indicating their subjective understanding and liking for the FOPLs. In the first stage of the survey, consumer demographic information was collected. The data were then analyzed at country level and as a cumulative sample.

The cumulative study sample is based on secondary data from extant study on 10 EU countries and on new primary data collected in an additional 10 EU member countries.

For the first group of 10 countries, the dataset was composed by evidence derived from four published studies [15,24,35,40], accumulating n = 3798 respondents, across Italy, France, Germany, Spain, Portugal, Greece, Romania, Poland, Slovenia, and the Netherlands. Additional primary data were collected on 762 additional paid respondents from 10 EU countries: Austria, Belgium, Czech Republic, Denmark, Estonia, Finland, Hungary, Ireland, Latvia, and Sweden. Participants were recruited through the Prolific platform. The regional distributions and the chronological presentation of the data collection are presented in Table 2.

Details of sample size for each country and socio-demographic information categorized by country are provided in Table 3.

## 3. Results

### 3.1. FOPL Performance—Descriptive Statistics by Country

Response data were analyzed through IBM SPSS Statistics, version 28 (SPSS Inc., Chicago, IL, USA). As shown in Table 1, we measured the subjective understanding with three constructs derived from pre-validated scales: comprehensibility, help to shop, and complexity reduction.

All reliability tests were consistent, showing a Cronbach’s alpha value higher than 0.70. The results for the 10 additional countries, where primary data were collected, are summarized in Table 4.

The overall *t*-test results of the aggregated answers from 20 EU countries indicate the subjective understanding and liking level. The NutrInform Battery reports a mean of M_NiB_ = 5.10 vs. Nutri-Score M_NS_ = 4.48 (t (4558) = −14.08, *p* < 0.01) in terms of comprehensibility; M_NiB_ = 5.01 vs. M_NS_ = 4.09 (t (4558) = −19.75, *p* < 0.01) for help to shop; M_NiB_ = 4.66 vs. M_NS_ = 3.75 (t (4558) = −18.47, *p* < 0.01) for complexity reduction; and M_NiB_ = 4.96 vs. M_NS_ = 4.78 (t (4558) = −4.26, *p* < 0.01) for liking.

From a by-country perspective, *t*-tests conducted in three countries are reported, while specific information for the other seven countries can be found in Table 5. Specifically, in Austria, the NutrInform Battery (vs. Nutri-Score) reports a mean of 5.01 vs. 4.79 (t (78) = 0.70, *p* = 0.49) for the comprehensibility; 4.79 vs. 4.20 (t (78) = 2.09, *p* = 0.04) for help to shop; 4.27 vs. 3.41 (t (78) = 3.37, *p* < 0.01) for complexity; and 4.73 vs. 5.19 (t (78) = 3.37, *p* = 0.12) for liking. In Belgium, the NutrInform Battery (vs. Nutri-Score) reports a mean of 5.01 vs. 5.46 (t (78) = −1.58, *p* = 0.12) for the comprehensibility; 4.57 vs. 4.44 (t (78) = 0.47, *p* = 0.64) for help to shop; 4.38 vs. 3.78 (t (78) = 2.02, *p* = 0.05) for complexity; and 4.72 vs. 5.61 (t (78) = −3.22, *p* < 0.01) for liking. In the Czech Republic (CR), the NutrInform Battery (vs. Nutri-Score) reports a mean of 4.93 vs. 5.01 (t (78) = −0.25, *p* = 0.80) for the comprehensibility; 4.70 vs. 4.18 (t (78) = 1.66, *p* = 0.10) for help to shop; 4.27 vs. 3.54 (t (78) = 2.69, *p* < 0.01) for complexity; and 4.75 vs. 5.30 (t (78) = −1.83, *p* = 0.07) for liking.

Compared to the Nutri-Score, NutrInform Battery is significantly more effective in improving comprehensibility in four countries, including Estonia, Finland, Hungary, and Ireland, and displays an overall higher comprehension in the remaining six countries. In terms of help to shop, NutrInform Battery shows a significantly higher score than Nutri-Score in eight countries while there are no significant differences of the two labels’ effectiveness in Belgium and the Czech Republic. Regarding complexity reduction of the FOPLs, NutrInform Battery shows superior performance to Nutri-Score across the additional 10 tested EU countries.

The above results confirm data from extant research on subjective understanding and liking [24], that displayed significantly higher scores for NutrInform Battery (vs. Nutri-Score) in comprehensibility (5.60 vs. 5.10, t (198) = 2.74, *p* < 0.01), help to shop (5.50 vs. 4.90, t (198) = 3.22, *p* < 0.01), complexity improvement (5.20 vs. 4.50, t (198) = 3.53, *p* < 0.01), and liking (5.60 vs. 5.10, t(198) = 2.85, *p* < 0.01).

The secondary data [15,35,40], with results of the between-subject experiment on how NutrInform Battery vs. Nutri-Score generates different levels of subjective understanding and liking across the ten countries are reported in Table S1. From the results, except for France which did not show significantly different results for comprehensibility, the NutrInform Battery label was considered more effective in improving subjective understanding on all its constructs of comprehensibility, help to shop, and complexity. In terms of liking, NutrInform Battery shows a significantly higher score vs. Nutri-Score in four countries, namely Italy, Greece, Portugal, and Slovenia, while in the Netherlands, Nutri-Score is more favored by participants.

Retrieving results from extant research on the other 10 EU countries, and based on secondary data [15,24,35,40], we can derive that NutrInform Battery is significantly more effective than Nutri-Score in improving subjective understanding on comprehensibility, help to shop, and complexity across nine EU countries.

### 3.2. FOPL-Country Interaction Effect

We validated our results using a between-subjects two-way ANCOVA for each dependent variable (comprehensibility, help to shop, complexity reduction, and liking), while including age, gender, education, occupation, and income as control variables. As presented in Table 6, we reported results starting from the three dimensions of subjective understanding and liking level. Specifically, we considered comprehensibility as the dependent variable and FOPL system (1  =  NutrInform Battery; 2  =  Nutri-Score), country and the interaction (FPOL * country), as independent variables while controlling for age, gender, education, occupation, and income.

The control variables did not show significant associations with comprehensibility except for education (F (1, 761) = 8.19, *p* =  0.004). The ANCOVA also showed that there is a significant main effect of NutrInform Battery on comprehensibility (M_NutrInform Battery_ = 5.14 vs. M_Nutri-Score_ = 4.79; F (1, 761) = 13.295, *p* <  0.001). Regarding the main effect of the country on the comprehensibility, it is reported to be non-significant (M_Austria_ = 4.86; M_Belgium_ = 5.30; M_CR (Czech Republic)_ = 5.00; M_Denmark_ = 4.91; M_Estonia_ = 4.75; M_Finland_ = 5.04; M_Hungary_ = 4.95; M_Ireland_ = 5.16; M_Latvia_ = 5.05; M_Sweden_ = 4.64; F (9, 761) = 1.644, *p* =  0.099). Further, there was a statistically significant interaction between the FOPL system and country on comprehensibility, whilst controlling for age, gender, occupation, and income (F (9, 761) = 2.838, *p* =  0.003).

Regarding help to shop as the dependent variable and FOPL system (1  =  NutrInform Battery; 2  =  Nutri-Score), country and the interaction (FOPL * country), as independent variables while controlling for age, gender, education, occupation, and income. The control variables did not show significant associations with help to shop except for age (F (1, 761) = 8.341, *p* =  0.004). The ANCOVA also showed that there is a significant main effect of NutrInform Battery on help to shop (M_NutrInform Battery_ = 4.94 vs. M_Nutri-Score_ = 3.96; F (1, 761) = 104.464, *p* <  0.001). Regarding the main effect of the country on help to shop, it is reported to be non-significant (M_Austria_ = 4.47; M_Belgium_ = 4.56; M_CR (Czech Republic)_ = 4.46; M_Denmark_ = 4.33; M_Estonia_ = 4.39; M_Finland_ = 4.58; M_Hungary_ = 4.47; M_Ireland_ = 4.67; M_Latvia_ = 4.54; M_Sweden_ = 4.04; F (9, 761) = 1.644, *p* =  0.099). Further, there was a statistically significant interaction between FOPL system and country on help to shop, whilst controlling for age, gender, occupation, and income (F (9, 761) = 2.48, *p* =  0.009).

Similarly, with complexity reduction as the dependent variable and FOPL system (1  =  NutrInform Battery; 2  =  Nutri-Score), country and the interaction (FOPL * country), as independent variables while controlling for age, gender, education, occupation, and income, the control variables did not show significant associations with complexity reduction except for occupation (F (1, 761) = 4.236, *p* =  0.04). The ANCOVA also showed that there is a significant main effect of NutrInform Battery on complexity reduction (M_NutrInform Battery_ = 4.55 vs. M_Nutri-Score_ = 3.50; F (1, 761) = 125.8, *p* <  0.001). Regarding the main effect of the country on complexity reduction, it is reported to be non-significant (F (9, 761) = 1.794, *p* =  0.066). Further, the interaction effect between FOPL system and country on complexity reduction is non-significant, whilst controlling for age, gender, occupation, and income (F (9, 761) = 1.16, *p* =  0.317).

Regarding liking the dependent variable and FOPL system (1  =  NutrInform Battery; 2  =  Nutri-Score), country and the interaction (FPOL * country), as independent variables while controlling for age, gender, education, occupation, and income, the control variables did not show significant associations with liking except for education (F (1, 761) = 3.954, *p* =  0.05). The ANCOVA also showed that there is no significant main effect of NutrInform Battery on liking (M_NutrInform Battery_ = 4.95 vs. M_Nutri-Score_ = 4.95; F (1, 761) = 0.006, *p* =  0.94). Regarding the main effect of the country on liking, it is reported to be non-significant (F (9, 761) = 1.715, *p* =  0.082). Further, there was a statistically significant interaction between FOPL system and country on liking, whilst controlling for age, gender, occupation, and income (F (9, 761) = 2.89, *p* =  0.002).

## 4. Discussion

This study investigates the comparative performance of NutrInform Battery and Nutri-Score in terms of subjective understanding and liking, two fundamental constructs in food decision making, and complement Pettigrew’s [9] analysis on objective understanding, in the perspective of large cumulative samples of consumers at EU level, contributing to the current research investigation on EU harmonization of FOPLs.

Evidence suggests that, based on a large, cumulated sample of 4560 respondents at aggregated level from 20 EU countries, NutrInform Battery achieves a significantly higher score on the three subjective understanding dimensions. At country level, NutrInform Battery is considered as superior in increasing consumer subjective understanding of nutritional knowledge and FOPL liking, compared to the Nutri-Score. With regards to specific constructs, in terms of comprehensibility, NutrInform Battery performs significantly better than Nutri-Score in 13 countries. In the other seven countries, the observed means of NutrInform Battery are constantly higher than Nutri-Score, though the differences are not statistically significant. From the perspective of help to shop, respondents exposed to NutrInform Battery show significantly higher scores in 18 countries. On complexity reduction, all respondents perceive NutrInform Battery to be more extensive and knowledgeable. In terms of liking, the cumulated result of the 20 EU countries shows that NutrInform Battery has a significantly higher score.

A by-country analysis highlights that respondents from Greece, Italy, Portugal, Slovenia, and Finland have a significantly higher level for NutrInform Battery, while participants from Belgium indicated higher scores for Nutri-Score. In the other countries, despite a higher liking level towards NutrInform Battery, results are not statistically significant. By further conducting cross-country analyses, we found that the liking level for the two FOPLs differs by country, showing a significant interaction effect. This element might be connected to the familiarity of consumers with the Nutri-Score, which in turn might influence consumer liking level toward the FOPL. Future research could also take familiarity as a control variable for FOPL research, considering that different EU member countries are promoting different FOPLs (e.g., Nutri-Score, NutrInform Battery, Keyhole, etc.).

In terms of control variables, our ANCOVA analysis revealed variations in effectiveness based on socio-demographic factors. Our results highlight the significant impact of education on comprehensibility and liking, age on help to shop, and occupation level on reducing complexity. These findings align with previous studies that emphasized the influence of age, education, and income level on the effectiveness of the two types of front-of-pack labels. Furthermore, our study contributes by demonstrating the additional socio-demographic aspect of occupation level.

Overall, evidence of this research showed a consistent superior effectiveness of NutrInform Battery in improving consumer subjective understanding, and an overall skew to superiority on liking, at EU level, complementing Pettigrew’s [9] analysis on objective understanding. To this end, the complementary results observed in this study confirm and emphasize the importance of considering both subjective and objective understanding when assessing the effectiveness of nutritional information systems. The Nutri-Score system, which usually performs better in terms of objective understanding, may provide clearer and more accurate information regarding the nutritional content of food products. This objectivity is likely to be advantageous for individuals who prefer a straightforward, standardized approach to evaluating food choices. On the other hand, the NutrInform Battery, despite its potential lower objective understanding scores, demonstrated superiority in terms of subjective understanding and confirmed the need for further research.

Given the sizeable and comparable robustness of the two studies on the effectiveness of FOPLs from two perspectives in the EU context, we propose that research should be continued, identifying new methods and new routes as a solid scientific foundation to support the choice of a harmonized FOPL.

In this logic, other research focused on exploring ways to bridge the gap between subjective and objective understanding in nutritional information systems [11,41]. For instance, one possible harmonized theoretical model, the front-of-pack acceptance model (FOPAM) [11,41], was introduced to explore relevant antecedents of behavioral intentions towards healthier and more informed food choices, including the exploration of constructs as front-of-pack labels’ easiness to use, usefulness, trust [11], attitude, and purchase intention. When consumers perceive front-of-pack nutritional labels as useful, credible, easy to use, and personally relevant, they are more likely to embrace and engage with the system, leading to more accepted food choices. The front-of-pack acceptance model might have contributed relevant insights to policymakers, food manufacturers, and public health organizations. In fact, it clarifies the fundamental role of consumer trust in the label, especially under the condition of algorithmic/computational information disclosure, and the relative acceptance of customers to different labels. By understanding the factors that influence front-of-pack nutritional label acceptance, stakeholders might then design and implement labeling systems that effectively communicate nutritional information and support consumers in making informed choices. Clear and credible labeling systems, combined with education and awareness campaigns, can enhance consumers’ understanding and utilization of front-of-pack labels, ultimately contributing to improved outcomes [11].

Alternatively, the effectiveness of combining two appropriate types of front-of-pack nutritional labels (e.g., NutrInform Battery and KeyHole) could be explored, beyond the current initial study [42,43], to understand the effects of combining front-of-pack nutritional labels for different typologies of EU consumers. Previous studies aimed to examine the impact of different types of front-of-pack nutritional label bundles on consumers’ subjective understanding and liking of the labels [11]. Consistent with previous literature, the complementary bundle of directive and nondirective labels outperformed non-complementary bundles of FOPLs, indicating higher utility for consumers [42,43]. This suggests that the presence of two complementary labels (directive and non-directive labels) strengthens the effects on subjective understanding and liking. This study revealed that the simultaneous presence of Keyhole (as a directive label) and NutrInform Battery (as a non-directive label) could improve subjective understanding.

Future research should also consider consumer characteristics and customized needs. For instance, eye tracking studies have shown that restrained consumers usually focus on calories information when exposed to FOP labels [44]. Future research could consider possible moderators in terms of FOPLs’ use, e.g., exploring how different types of consumers use front-of-pack nutritional labels and under what conditions consumers use FOPLs as guidance. Different demographic groups may have varying levels of familiarity with nutritional concepts, varying levels of health consciousness, and distinct cultural dietary practices. Understanding these differences can inform the design and presentation of nutritional information systems, enabling them to be more effective in different contexts and for diverse populations.

While the current study has provided valuable insights into the comparison between subjective and objective understanding of front-of-pack nutritional labels, there are several avenues for future research to expand upon these findings and further enhance our understanding of the topic. Adopting a longitudinal research design would be beneficial to examine the stability of subjective and objective understanding over time. By following participants’ understanding of front-of-pack labels over an extended period, researchers can assess whether any changes occur in their perceptions and preferences. This would provide insights into the long-term effectiveness and impact of different labeling systems on consumers’ understanding and decision making. Supplementing the quantitative data with qualitative research methods, such as focus groups or interviews, can provide a deeper understanding of consumers’ perceptions and experiences regarding front-of-pack labels. Qualitative research allows for the exploration of the underlying reasons and motivations behind consumers’ subjective and objective understanding. It can uncover rich insights into the factors that influence their preferences, decision-making processes, and the contextual nuances associated with their understanding of front-of-pack labels. Future research could delve into comparative analyses of different front-of-pack labeling systems, beyond Nutri-Score and NutrInform Battery. Conducting research in real-world settings, such as supermarkets or online shopping platforms, can provide valuable insights into how consumers interact with front-of-pack labels in their natural decision-making environment. By observing consumers’ actual behaviors and choices, researchers can gain a deeper understanding of the practical implications of subjective and objective understanding on purchasing decisions and overall dietary patterns.

## 5. Conclusions

The importance of considering different and complementary elements of consumers’ decision-making processes in food appear relevant, as performances and superiority might differ significantly, and considering only one antecedent aspect of decision making would cast a partial view of the reality.

On the one hand, Pettigrew [9], via a large, cumulated sample of respondents, highlighted the superiority of Nutri-Score on eight EU countries, regarding objective understanding. On the other hand, this study, through a cumulated large sample of respondents in 20 countries demonstrated that NutrInform Battery is more effective than Nutri-Score in improving both consumer subjective understanding and liking among European consumers.

Based on diverging results derived from two large samples, that analyzed complementary elements of the food decision making process, policymakers should then further encourage research to address the issue of identifying the best FOPL to support customers toward healthier and more informed food choices.

## Figures and Tables

**Table 1:** Measurement items for subjective understanding and liking (7-point Likert Scale).

Comprehensibility [37]	I feel well informed by the food label
	This label is believable and trustworthy
	This label is easy to interpret
Help to shop [37]	This label helps me to understand the product composition
	This label helps me to understand different nutritional values
	This label makes it easier to choose food
Complexity reduction [37]	The food label is rather extensive
	Using this food label to choose foods is better than just relying on my knowledge about what is in them
Liking [38]	How do you evaluate the label?
	Answers with “Bad/ good”, “unfavorable/favorable”, and “negative/ positive”

**Table 2:** An overview of the process of study execution across EU member countries.

	Countries	Participants Number
Study 1	Italy	200
Study 2	France, Germany, Greece, Italy, Portugal, Romania, Spain	2776
Study 3	Poland	424
Study 4	Slovenia and the Netherlands	398
Study 5 *	Austria, Belgium, Czech Republic, Denmark, Estonia, Finland, Hungary, Ireland, Latvia, and Sweden	762

* New sample of primary data collected for this paper.

**Table 3:** Sociodemographic of the additional population sample, by country, n (%).

Country (N = Participants Number)	Austria		Belgium		Czech Republic		Denmark		Estonia	
	N = 80		N = 80		N = 80		N = 53		N = 80	
	Group 1 (NutrInform Battery)	Group 2 (Nutri-Score)	Group 1 (NutrInform Battery)	Group 2 (Nutri-Score)	Group 1 (NutrInform Battery)	Group 2 (Nutri-Score)	Group 1 (NutrInform Battery)	Group 2 (Nutri-Score)	Group 1 (NutrInform Battery)	Group 2 (Nutri-Score)
**Variables**	**(%)**	**(%)**	**(%)**	**(%)**	**(%)**	**(%)**	**(%)**	**(%)**	**(%)**	**(%)**
**Age**										
18–24	28.20	39.00	32.70	53.10	45.20	55.20	34.20	28.60	26.20	47.40
25–34	43.60	41.50	40.80	21.90	40.50	29.00	36.80	38.10	52.40	34.20
35–49	23.10	14.60	22.40	21.90	11.90	13.20	23.70	23.80	21.40	18.40
50+	5.10	4.90	4.10	3.10	2.40	2.60	5.30	9.60	0.00	0.00
**Gender**										
Men	45.00	54.00	58.00	34.40	38.10	68.40	44.00	58.00	45.20	58.00
Women	55.00	46.00	42.00	65.60	61.90	31.60	56.00	42.00	54.80	42.00
**Country (N = Participants number)**	**Finland**		**Hungary**		**Ireland**		**Latvia**		**Sweden**	
	**N = 70**		**N = 80**		**N = 80**		**N = 80**		**N = 79**	
	Group 1 (NutrInform Battery)	Group 2 (Nutri-Score)	Group 1 (NutrInform Battery)	Group 2 (Nutri-Score)	Group 1 (NutrInform Battery)	Group 2 (Nutri-Score)	Group 1 (NutrInform Battery)	Group 2 (Nutri-Score)	Group 1 (NutrInform Battery)	Group 2 (Nutri-Score)
**Variables**	**(%)**	**(%)**	**(%)**	**(%)**	**(%)**	**(%)**	**(%)**	**(%)**	**(%)**	**(%)**
**Age**										
18–24	36.80	23.80	40.50	39.50	12.20	23.10	51.00	54.90	8.90	14.70
25–34	31.60	45.20	42.80	42.10	22.00	15.40	32.70	41.90	55.60	55.90
35–49	23.70	26.20	14.30	18.40	48.80	46.10	14.30	3.20	17.80	14.70
50–64	5.30	4.80	2.40	0.00	14.60	10.30	2.00	0.00	15.50	14.70
65+	2.60	0.00	0.00	0.00	2.40	5.10	0.00	0.00	2.20	0.00
**Gender**										
Men	38.00	61.90	50.00	50.00	48.80	54.00	57.00	35.00	58.00	58.80
Women	62.00	38.10	50.00	50.00	51.20	45.00	43.00	65.00	42.00	41.20

**Table 4:** Reliability test for dependent constructs (Cronbach alphas).

	Austria	Belgium	Czech Republic	Denmark	Estonia	Finland	Hungary	Ireland	Latvia	Sweden
Comprehensibility	0.827	0.777	0.856	0.822	0.818	0.849	0.780	0.814	0.759	0.829
Help to shop	0.710	0.790	0.772	0.734	0.780	0.852	0.744	0.782	0.737	0.865
Complexity reduction	0.715	0.701	0.744	0.721	0.780	0.791	0.747	0.783	0.756	0.761
Liking level	0.936	0.893	0.926	0.887	0.874	0.919	0.890	0.927	0.851	0.926

**Table 5:** *t*-test regarding the subjective understanding and the liking level by country.

	Austria				Belgium				Czech Republic (CR)				Denmark				Estonia			
	NIB	NS	*t*-Value	*p*	NIB	NS	*t*-Value	*p*	NIB	NS	*t*-Value	*p*	NIB	NS	*t*-Value	*p*	NIB	NS	*t*-Value	*p*
Comprehensibility	5.01	4.79	0.70	0.49	5.01	5.46	−1.58	0.12	4.93	5.01	−0.25	0.80	4.82	5.02	−0.58	0.56	5.15	4.39	2.54	0.01
Help to shop	4.79	4.20	2.09	0.04	4.57	4.44	0.47	0.64	4.70	4.18	1.66	0.10	4.78	3.86	2.45	0.02	5.01	3.79	4.40	<0.01
Complexity reduction	4.27	3.41	3.37	<0.01	4.38	3.78	2.02	0.047	4.27	3.54	2.69	<0.01	4.04	3.17	2.30	0.03	4.83	3.50	5.11	<0.01
Liking level	4.73	5.19	3.37	0.12	4.72	5.61	−3.22	<0.01	4.75	5.30	−1.83	0.07	4.53	4.78	−0.71	0.48	5.04	4.65	1.31	0.20
	Finland				Hungary				Ireland				Latvia				Sweden			
	NIB	NS	*t*-value	*p*	NIB	NS	*t*-value	*p*	NIB	NS	*t*-value	*p*	NIB	NS	*t*-value	*p*	NIB	NS	*t*-value	*p*
Comprehensibility	5.71	4.37	4.40	<0.01	5.36	4.60	2.80	<0.01	5.55	4.85	2.64	0.01	5.26	4.86	1.63	0.11	4.67	4.61	0.19	0.85
Help to shop	5.52	3.64	5.80	<0.01	5.21	3.78	5.78	<0.01	5.30	4.11	4.25	<0.01	5.95	4.15	2.79	0.01	4.64	3.45	3.29	<0.01
Complexity reduction	4.88	3.36	4.72	<0.01	4.68	3.67	3.88	<0.01	4.73	3.73	4.00	<0.01	4.67	3.69	3.89	<0.01	4.62	3.18	4.84	<0.01
Liking level	5.46	4.52	2.90	<0.01	5.34	4.84	1.94	0.06	5.30	5.01	1.01	0.32	5.10	5.12	−0.06	0.95	4.54	4.63	−0.24	0.81
Note: NutrInfom Battery = NIB; Nutri-Score = NS																				

**Table 6:** Results of the two-way analysis of covariance (ANCOVA).

Predictor	Comprehensibility						Help to Shop						Complexity						Liking					
	SS	df	MS	F	*p*	η2	SS	df	MS	F	*p*	η2	SS	df	MS	F	*p*	η2	SS	df	MS	F	*p*	η2
FOPL (A)	22.477	1	22.477	13.295	<0.001	0.018	177.09	1	177.09	104.464	<0.001	0.124	185.791	1	185.791	125.8	<0.001	0.146	0.01	1	0.01	0.006	0.939	0
Country (B)	25.01	9	2.779	1.644	0.099	0.02	20.905	9	2.323	1.37	0.198	0.016	23.839	9	2.649	1.794	0.066	0.021	26.726	9	2.97	1.715	0.082	0.021
FOPL * Country (A * B)	43.185	9	4.798	2.838	0.003	0.033	37.838	9	4.204	2.48	0.009	0.029	15.438	9	1.715	1.161	0.317	0.014	45.001	9	5	2.887	0.002	0.034
Gender	0.757	1	0.757	0.448	0.504	0.001	1.917	1	1.917	1.131	0.288	0.002	1.353	1	1.353	0.916	0.339	0.001	2.06	1	2.06	1.189	0.276	0.002
Age	6.291	1	6.291	3.721	0.054	0.005	14.14	1	14.14	8.341	0.004	0.011	4.473	1	4.473	3.029	0.082	0.004	2.233	1	2.233	1.29	0.256	0.002
Education	13.849	1	13.849	8.191	0.004	0.011	2.673	1	2.673	1.577	0.21	0.002	0.759	1	0.759	0.514	0.474	0.001	6.848	1	6.848	3.954	0.047	0.005
Occupation	0.045	1	0.045	0.026	0.871	0	0.675	1	0.675	0.398	0.528	0.001	6.256	1	6.256	4.236	0.04	0.006	1.023	1	1.023	0.591	0.442	0.001
Income	0.515	1	0.515	0.305	0.581	0	5.299	1	5.299	3.126	0.077	0.004	0.042	1	0.042	0.028	0.866	0	0.204	1	0.204	0.118	0.732	0
Error	1362.218	761					1511.765	761					1351.267	761					1365.303	761				
Comprehensibility: R^2^ = 0.085; Help-to-shop: R^2^ = 0.174; Complexity: R^2^ = 0.194; Liking: R^2^ = 0.065																								
SS: sum of squares; df: degrees of freedom; MS: mean square; F: F-test; η^2^: effect size.																								
Age, gender, level of education, occupation, and income are covariates. * means the interaction effect between the two variables.																								

## Data Availability

Not applicable.

## Supplementary Materials

The following supporting information can be downloaded at: https://www.mdpi.com/article/10.3390/nu15132852/s1, Figure S1: Template of front-of-pack labels in the comparative studies. Table S1: Secondary data across 10 EU countries: *t*-test of subjective understanding and liking level [15,34,35,39]. Table S2: Obesity Rate in the 20 EU member countries.
