# Supplementary material for: A 20-Country Comparative Assessment of the Effectiveness of Nutri-Score vs. NutrInform Battery Front-of-Pack Nutritional Labels on Consumer Subjective Understanding and Liking
Source: Nutrients. 2023 Jun 23;15(13):2852. doi: 10.3390/nu15132852 (PMC10343458; doi:10.3390/nu15132852)
Supplement: Supplementary file 1 [file nutrients-15-02852-s001.zip › nutrients-2441827-supplementary.pdf]

Table S1. Secondary data across 10 EU countries: T-test of subjective understanding and liking level.

	Italy (N=200)				France (N=341)				Germany (N=330)				Greece (N=440)				Italy (N=368)			
	NIB	NS	T-value	P	NIB	NS	T-value	P	NIB	NS	T-value	P	NIB	NS	T-value	P	NIB	NS	T-value	P
Comprehensibility	5.60	5.10	2.74	<0.01	4.92	4.62	1.84	0.07	5.20	4.60	3.31	<0.01	5.08	4.06	7.20	<0.01	5.06	4.40	4.09	<0.01
Help to shop	5.50	4.90	3.22	<0.01	4.82	4.27	3.15	<0.01	5.08	4.23	4.80	<0.01	4.88	3.84	7.04	<0.01	5.09	4.09	5.78	<0.01
Complexity	5.20	4.50	3.53	<0.01	4.67	4.07	3.37	<0.01	4.80	4.20	3.09	<0.01	4.50	3.50	6.84	<0.01	4.81	3.78	5.78	<0.01
Liking level	5.60	5.10	2.85	<0.01	4.89	5.05	1.03	0.30	4.80	4.95	0.70	0.48	4.77	4.48	2.18	<0.05	5.07	4.47	3.50	<0.01
	Portugal (N=417)				Romania (N=440)				Spain (N=440)				Poland (N=424)				Slovenia (N=153)			
	NIB	NS	T-value	P	NIB	NS	T-value	P	NIB	NS	T-value	P	NS	NIB	T-value	P	NS	NIB	T-value	P
Comprehensibility	5.02	4.24	7.03	<0.01	5.35	4.84	3.51	<0.01	5.03	4.58	3.02	<0.01	4.44	5.27	-5.60	<0.01	3.27	4.92	-7.46	<0.01
Help to shop	5.10	3.90	8.50	<0.01	5.40	4.80	4.46	<0.01	5.06	4.20	5.57	<0.01	4.46	5.34	-5.47	<0.01	3.37	5.00	-7.21	<0.01
Complexity	4.60	3.50	7.52	<0.01	5.07	4.20	4.73	<0.01	4.65	3.76	5.46	<0.01	4.21	5.16	-5.72	<0.01	2.52	3.99	-6.07	<0.01
Liking level	5.04	3.45	11.02	<0.01	5.30	5.20	0.80	0.42	4.95	4.70	1.58	0.12	4.96	5.12	-1.15	0.13	4.50	4.96	-2.61	0.01
	The Netherlands (N=245)																			
	NS	NIB	T-value	P																
Comprehensibility	4.31	4.98	-3.80	<0.01	**Note**: NutrInfom Battery=NIB; Nutri-Score=NS [15, 24, 35, 40]															
Help to shop	2.75	4.41	-8.86	<0.01																
Complexity	2.43	3.59	6.43	<0.01																
Liking level	4.41	4.32	-4.17	0.01																

Figure S1. Template of front-of-pack labels in the comparative studies

Nutri Score label template

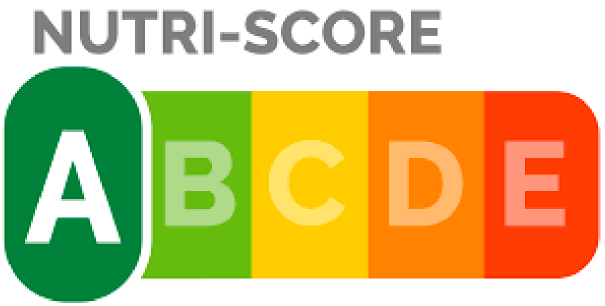

NutrInform Battery label template

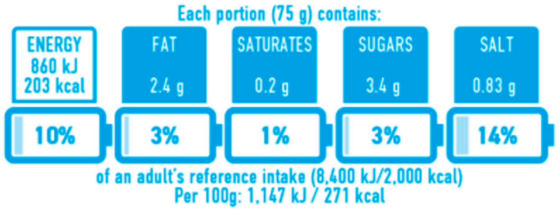

Table S2. Obesity Rate in the 20 EU member countries

Country	Austria	Belgium	Czech Republic	Denmark	Estonia	Finland	France	Germany	Greece	Hungary
Obesity level	52.20%	50.20%	60.00%	50.40%	56.70%	59.00%	47.20%	53.50%	57.60%	59.90%
Country	Ireland	Italy	Latvia	the Netherlands	Poland	Portugal	Romania	Slovenia	Spain	Sweden
Obesity level	54.40%	45.70%	58.30%	50.00%	58.10%	55.90%	58.70%	58.10%	53.70%	51.30%
